# Autocratic Legalism, Partisanship, and Popular Legitimation in Authoritarian Cameroon

**DOI:** 10.1093/poq/nfad051

**Published:** 2023-12-12

**Authors:** Natalie Wenzell Letsa, Yonatan L Morse

**Affiliations:** Assistant Professor, Department of International and Area Studies, University of Oklahoma, Norman, OK, US; Associate Professor, Department of Political Science, University of Connecticut, Storrs, CT, US

## Abstract

Authoritarian regimes regularly turn to the law to justify repression. This article examines whether invoking legal institutions has a persuasive effect on public perceptions of repression, and whether that effect is shaped by partisanship. The article uses the case of Cameroon’s Special Criminal Tribunal, created in 2011 to prosecute high-profile corruption cases. A survey experiment was designed that describes the arrest and trial of a suspected corrupt oppositional minister and reminds a treatment group about the Special Criminal Tribunal. The results show that neither regime nor opposition partisans are swayed by legal justifications for repression. By contrast, nonpartisans respond negatively to autocratic legalism, particularly those with low levels of regime trust. The article clarifies when autocratic legalism might be used for public legitimation, suggests that partisanship is a useful lens for understanding public opinion in an autocracy, and elaborates upon the meaning of nonpartisanship in electoral authoritarian regimes.

Growing attention has been given to the role of constitutions, law, and courts in sustaining authoritarian rule (de Sa e Silva 2022). Referred to interchangeably as “state lawfare” (Gloppen 2018), “autocratic legalism” (Corrales 2015; Schepple 2018), or “rule by law” (Ginsburg and Moustafa 2008), this literature complements other work on the role of quasi-democratic regimes in sustaining autocracy. These issues are especially relevant in the context of sub-Saharan Africa, where scholars have noted an increase in the use of autocratic legalism. For instance, in 2015 Tanzania’s ruling party rushed through a cybersecurity law ostensibly meant to combat online misinformation, but in reality used to persecute civil society activists. In 2021, Senegal passed an antiterror law on the grounds of fighting extremism, which has drawn criticism for allowing the government to widely define political speech as terrorism. In 2017, Zimbabwe created a new specialized anticorruption court that has been accused of selective application of the law.

While scholarship notes several functions of autocratic lawmaking, it fundamentally involves some element of legitimation (Whiting 2017). By using the façade of law, autocrats hope to engage in repressive behavior while limiting political and social opposition. However, we know comparatively little about which audience autocrats seek legitimation from and to what extent autocratic legalism impacts public opinion regarding repressive behavior. In a domestic context, does autocratic legalism work uniformly, or are its effects heterogeneous and mitigated by individual-level factors? These questions are difficult to answer decisively given the opaqueness of authoritarian regimes and the inherent challenges in soliciting credible public opinion information regarding sensitive issues.

This article explores how citizens respond to autocratic legalism, and how partisan orientations shape that reaction. Although partisanship is a key lens for understanding public opinion in regimes with competitive elections—both inside and outside of Africa—it has only recently been applied specifically to public opinion in *autocratic regimes* (Frye and Borisova 2019; Letsa 2019; Reuter 2021). Most work on public opinion in autocratic regimes relies on education and socioeconomic status to explain variation in political beliefs (Lipset 1959; Croke et al. 2016; Letsa and Wilfahrt 2018). Further, while the handful of studies on partisanship in autocracies have looked at differences between ruling-party partisans and opposition partisans, there is almost no literature on nonpartisan beliefs in autocratic contexts. But this is a question at the heart of autocratic legalism: If legal justifications are intended to legitimate repression for domestic audiences, is the strategy geared toward core regime supporters, core opposition supporters, or unaffiliated voters?

We answer this question in the context of Cameroon—one of Africa’s longest-standing autocracies. In 2011, as part of a broader anticorruption endeavor, the Special Criminal Tribunal (SCT) was created to prosecute high-profile corruption cases. However, the SCT has come under scrutiny for its use as a political tool that targets the president’s adversaries. To test the effect of autocratic legalism on public opinion, we designed a survey experiment embedded within an original survey fielded in four regions of Cameroon. The survey experiment described a realistic scenario of possible repression—the prosecution of an oppositional minister on corruption charges—and told a treatment group that it was the SCT that prosecuted the case. The effect of this legal justification was then evaluated by partisan affiliation, whether regime, opposition, or nonpartisan.

A priori, we hypothesized that regime partisans would react most positively to autocratic legalism and opposition partisans would react most negatively. This is consistent with work that indicates that regime supporters are sensitive to regime repression, and therefore most susceptible to forms of persuasion (Peisakhin and Rozenas 2018; Reuter and Szakonyi 2021). By contrast, opposition partisans are more distrustful of legal institutions and therefore may reduce their support for repression when explicitly reminded of them. Our results did not provide support for these expectations: although ruling-party partisans were the most supportive of repression and opposition partisans were the least supportive, neither group in our survey sample had a reaction to autocratic legalism. Both groups likely have such strong prior opinions about the regime that exposure to legalism does nothing to sway those beliefs.

Given the scarcity of theoretical work on nonpartisanship in autocracies, it was unclear, a priori, how they might respond to autocratic legalism. We therefore only provide post hoc exploratory analysis of the experimental data for the nonpartisan subgroup. It is possible that most nonpartisans are largely apolitical, in which case they may on average not react one way or another toward autocratic legalism. Alternatively, nonpartisans may be “closeted partisans,” who in fact hold political beliefs consistent with either ruling-party or opposition partisans and will respond similarly (Keith et al. 1992). However, against our expectations, nonpartisans in our sample react *negatively* to autocratic legalism; they are much less likely to accept autocratic repression when primed to think of the SCT. This undermines evidence for the notion that nonpartisans are largely apolitical and persuadable. When split into pro- and antiregime leaning groups, important differences emerge. Pro-regime nonpartisans do in fact mirror overt regime partisans and demonstrate high levels of support for repression but no response to autocratic legalism. By contrast, antiregime nonpartisans are not quite like overt opposition partisans. They are more likely to support regime repression but respond negatively to exposure to autocratic legalism.

Because of the lack of prior theorization about the ideological nature of nonpartisanship in electoral autocracies, we only offer post hoc interpretation of our results. We conjecture that nonpartisanship might hold a different meaning in electoral autocracies. Many nonpartisans might not be just antiregime, but anti-establishmentarian. Like opposition partisans they mistrust the regime, but they further mistrust the entire political system. Consequently, they are more likely to approve of the repression of potentially corrupt political figures, but also disapprove strongly of the use of questionable legal institutions to do so. More testing is needed to confirm these findings.

The article provides some of the first evidence regarding the role of partisanship in understanding public opinion about autocratic legalism. The evidence, while limited by the constraints of research in an authoritarian setting, suggests that autocratic legalism has very minimal domestic effects, at least in legitimizing repression of elites. Overt partisanship strongly determines how individuals view regime behavior, and therefore limits the effects of autocratic legalism. Only the opinions of the nonpartisans in our sample are moved by invoking legal institutions, but not in the intended direction. To the chagrin of a dictator, invoking autocratic legalism appears to *delegitimize* political repression. These findings contribute to our understanding of the aims of autocratic legalism and their actual effect on domestic public opinion, while shedding new light on the nature of nonpartisanship in electoral autocracies.

## Why Do Authoritarian Regimes “Rule by Law”?

The use of legal institutions to curtail political opposition is not new in authoritarian regimes (Hendersone 1991). Historically, however, legal innovations were analyzed primarily for their coercive ends, with less attention given to the comparative benefits of using judicial versus extrajudicial forms of repression. More recent scholarship on autocratic lawmaking seeks to better understand how autocratic legalism might serve various functions in an autocracy, such as legitimation, social control, counterbalancing other institutions, and monitoring administrative agents (Ginsburg and Moustafa 2008, pp. 4–5). The increased attention to autocratic legalism is also driven by the troubling proliferation of controversial new laws and judicial bodies in contemporary authoritarian settings. Thus, over the past decade we have witnessed a proliferation of new laws that regulate the right to protest (Goodfellow 2014), the funding of NGOs (Dupuy, Ron, and Prakash 2016), and the use of cyberspace (Frantz, Kendall-Taylor, and Wright 2020).

While legitimation is not the only function of autocratic lawmaking, it is generally viewed as its most central feature (Whiting 2017). A central question is: Whom do autocrats seek this legitimation from? On the one hand, Fiona Shen-Bayh examines how judicial repression is useful for containing threats from within ruling coalitions (Shen-Bayh 2018). On the other hand, autocratic legalism might primarily address scrutiny from international actors. Autocrats might create legal institutions that align the regime with global norms, and consequently shield it from international scrutiny (Arriola, Rakner, and Van de Walle 2023). For instance, in response to donor requests many authoritarian regimes developed special prosecutorial devices to ostensibly combat corruption that could also be used to target opponents with little pushback. Similarly, national security laws meant to align regimes with the global war on terror could be turned against domestic opponents (Morse 2019).

Critical to our inquiry is whether autocratic legalism involves legitimation in the face of ordinary citizens. If acts of repression are justified by quasi-legal means, does it reduce domestic opposition to said repression, and if so, from which groups? The existing scholarship has not addressed this question specifically but highlights the importance of considering prior beliefs in understanding public opinion. Foundational work on Brazil’s military regime suggests that citizens with proregime beliefs are more susceptible to government propaganda (Geddes and Zaller 1989). Similarly, Hannah Chapman shows how Russian citizens who trust President Putin are more receptive to government communication strategies (2021). Other recent survey work from Russia shows how core supporters are particularly sensitive to overt repression (Reuter and Szakonyi 2021). We extend this framework to the topic of autocratic legalism and explore a wider range of prior beliefs by using the comparatively underused lens of partisanship.

## The Role of Partisanship in Electoral Autocracies

Our analysis focuses on partisanship as a key lens for understanding public opinion in electoral autocracies. Just as in democratic regimes, citizens in electoral autocracies can choose to support a political party—to identify as partisans—or not, opting instead for (or defaulting to) nonpartisanship. Partisans in electoral autocracies can be split into two main subtypes: ruling-party partisans and opposition partisans. Ruling-party partisans, of course, support the regime in power. Strong partisans may vote regularly, attend local party meetings, or even do significant work such as organize or campaign for the party. Ideologically, ruling-party partisans are more likely to believe that the regime is democratic, and hold more favorable views of the country (Carlson 2016; Letsa 2019). For example, looking at the electoral autocracies in Round 6 of the Afrobarometer,^1^ citizens who report feeling close to the ruling party, on average, rated their country as being “a democracy, but with minor problems,” whereas all other respondents ranked their country closer to being “a democracy, but with major problems” (2.96 versus 2.34 on a four-point scale). Ruling-party partisans consistently gave higher ratings than everyone else to their assessments of how the government was handling the economy (2.46 versus 2.00), reducing crime (2.73 versus 2.37), and fighting corruption (2.10 versus 1.79). However, since in electoral autocracies affiliation with the ruling party is often key for economic mobility (Rosenfeld 2020), some regime partisans may not necessarily hold their beliefs strongly.

Inversely, since affiliation with an opposition party in authoritarian regimes is risky, they tend to draw individuals with highly distinct ideological convictions (Greene 2007; Weghorst 2022). Opposition partisans are much more likely to believe that the regime is autocratic, repressive, and corrupt, as well as hold more pessimistic views of the economy (Carlson 2016; Letsa 2019). Importantly, opposition partisanship may have different meanings in regimes without an independent opposition, but we consider an independent opposition a scope condition to our theory.

Although some work has been done to understand these two distinct groups of partisans in autocracies, very little scholarship has investigated nonpartisans. In the context of democratic regimes, it has become paradigmatic to think of independent or nonpartisan voters as “closeted partisans” (Keith et al. 1992). Although they report themselves to be unaffiliated with any party, their political beliefs usually align strongly with one or another. More recently, Klar and Krupnikov (2016) have argued that nonpartisans differ primarily in terms of their proclivity to engage in politics. Most studies of nonpartisanship in Africa are focused on which campaign strategies are most effective in mobilizing these voters, rather than their political beliefs (Ishiyama and Fox 2006; Weghorst and Lindberg 2013). However, nearly all the work done on partisanship in Africa is in the context of democratic regimes, specifically Ghana and Kenya, or using cross-national data from the early rounds of the Afrobarometer, which primarily sampled in Africa’s more democratic regimes.

Given the lack of theorizing about nonpartisans under authoritarianism, we propose two different possibilities for conceptualizing nonpartisanship. First, nonpartisans may be truly apolitical. They are not partisans because they have little interest in politics and are largely uninformed about most political issues. Given the futility of effecting change through voting or other political actions—as well as the heightened risk—it is plausible that many citizens of autocracies choose to simply disengage from the system altogether. Thus, nonpartisans may be risk-averse citizens who are pointedly uninterested in politics and hold fewer political opinions in general.

Alternatively, like in advanced democracies, nonpartisans may be “closeted partisans.” Although they may say that they do not feel close to any political party, they could in fact look ideologically similar to either ruling-party or opposition partisans. While there are a host of beliefs that separate ruling-party partisans from opposition partisans, in the context of electoral autocracies we propose that one of the most consistent dividers should be trust in the president. In such regimes, presidents are not just the head of the party in power, but also a national symbol of the regime itself. Thus, if anything should divide nonpartisan regime leaners from opposition leaners, it should be the most visible and well-known political figure in the country. Round 6 of the Afrobarometer data indicates that ruling-party and opposition partisans are firmly divided on the question of trust in the president and ruling party partisans trust the president much more than do opposition partisans (2.53 versus 1.29). Indeed, this is the largest trust gap of all political institutions, apart from the ruling party itself. Nonpartisans fall exactly in the middle (1.88).^2^ The following section theorizes how these different groups—ruling party partisans, opposition partisans, and nonpartisans—may react differently to legal justifications for repression.

## Partisanship and the Response to Autocratic Legalism

The central question of this article is whether the invocation of autocratic legalism can change public perceptions of regime behavior, and whether this effect is conditioned by partisanship. In the context of competitive elections, there is now a large literature that has demonstrated the role of partisan-motivated reasoning in assessing government performance (Bartels 2002; Taber and Lodge 2006; Gerber and Huber 2010), supporting illicit behavior like corruption (Anduiza, Gallego, and Munõz 2013; Blais, Gidengil, and Kilibarda 2017), and antidemocratic lawmaking (Ahlquist et al. 2018; Graham and Svolik 2020). Our expectation is that due to this bias, partisans are likely to respond to autocratic legalism in opposite directions. Thus, ruling-party partisans will be more likely to approve of potentially repressive acts when they are justified by legal institutions, since it mitigates their concern with the overt use of repression. Inversely, opposition partisans will be less likely to approve of repression when legal institutions are invoked because such institutions are viewed in a negative light. Given the ambiguity of the repression and the endemic nature of corruption in Cameroon, opposition partisans may be willing to support the prosecution of a corrupt leader—but would be less inclined if the prosecution was conducted under the auspices of a notoriously politicized legal institution.

*Hypothesis 1: Reminding ruling-party partisans of autocratic legalism will increase support for autocratic behavior.*

*Hypothesis 2: Reminding opposition partisans of autocratic legalism will decrease support for autocratic behavior.*

We acknowledge that partisans might not be moved by legal justifications for repression. If both groups begin from comparatively higher (lower) levels of support for regime behavior, it may be difficult to detect the effects of autocratic legalism on such support. In other words, their baseline acceptance (rejection) of repression could mean that invoking a legal justification for the repression is unlikely to make them accept (reject) repression even more. For this reason, we deliberately designed a prompt where the repression is not blatantly severe, such as the use of physical violence against ordinary citizens.

For nonpartisans, there is no clear hypothesis regarding their behavior, which depends on which model of nonpartisanship is prevalent. Instead, we present some post hoc exploratory expectations for nonpartisans given these two different models. In the first model, nonpartisans are apolitical and do not hold very strong political opinions. As a result, invoking legal institutions may have little effect on their overall approval of repressive acts. Apolitical nonpartisans may not have heard of such institutions or understand their history or meaning. If this first scenario is correct, we would expect to find no overall treatment effect among nonpartisans. The second model predicts that nonpartisans are actually “closeted partisans.” Although they do not explicitly support any specific political party, they do in fact hold political beliefs that are consistent with those of partisans. Thus, if nonpartisans are actually “closeted partisans,” we would expect them to divide naturally into “ruling-party leaners” and “opposition leaners.” We measure this divide using responses to the question “How much do you trust the president of the Republic?” In our sample of Cameroonians who responded to the core survey experiment, 235 nonpartisans reported that they trusted the president “somewhat” (71) or “a lot” (164) and were thus coded as ruling-party leaners. The other 179 nonpartisans said they trusted him “just a little” (76) or “not at all” (103) and were thus coded as opposition leaners. If the second model of nonpartisanship holds, then we would expect that nonpartisans who trust the president will react to autocratic legalism in the same way that ruling-party partisans do, and those who do not trust the president will react in the same way as opposition partisans.

## Research Design

### Case Selection—Cameroon

We use data from Cameroon, one of the oldest autocratic regimes in the world. Cameroon gained independence from France and Britain in 1960–1961 under the leadership of President Ahmadou Ahidjo. Within five years of independence, Ahidjo consolidated all of Cameroon’s political parties under the umbrella of the *Union nationale camérounaise* (UNC) and outlawed opposition parties. In 1982, Ahidjo handed power over peacefully to his hand-picked successor, Paul Biya, who renamed the UNC the *Rassamblement démocratique du people camérounaise* (RDPC) in 1985. In 1990, under immense pressure from sustained protest movements, Biya was forced to legalize opposition parties. Despite close competition early on, Biya and the RDPC have come to dominate Cameroonian elections. The dubious integrity and lopsided outcomes of these elections have made Cameroon an established electoral autocracy.

Consequently, the meaning of partisanship in Cameroon corresponds with our theoretical framework. The ruling party has core constituencies, primarily in the Centre, South, and East regions, but regularly wins most of the vote in every region of the country. Further, just like in other electoral autocracies, support for the ruling party is a gateway to economic advancement. By contrast, opposition support is limited due to harassment and increasing fragmentation. Historically the most important opposition party was the Social Democratic Front (SDF), based primarily in Cameroon’s English-speaking regions. In recent years the SDF has lost ground, and the main opposition is driven by the *Mouvement pour la Rennaisance du Cameroun* (MRC). Alongside the SDF and MRC are several other small parties that have either been co-opted or only mobilize small segments of the population. In this environment, few people truly believe the opposition can win power through the ballot box, yet most of these opposition parties maintain a cadre of committed voters.

Within this context, the regime has employed new legal mechanisms to constrain and disempower opposition. We focus specifically on the use of special courts to prosecute corruption. In 2004, with donor support, Cameroon launched *Opération épervier* (Operation Sparrowhawk) to prosecute cases of public embezzlement identified by a new national anticorruption commission. As part of this initiative, several high-ranking individuals were convicted for lengthy prison terms in Cameroon’s High Court. Many of these trials were perceived as politically motivated to eliminate Biya’s political rivals.^3^ In 2011, the Special Criminal Tribunal (SCT) was founded to selectively prosecute high-profile cases of embezzlement in excess of CFA 50 million ($90,000). The SCT was also ostensibly intended to streamline the legal process and address criticism regarding the politicization of Operation Sparrowhawk. Yet President Biya still retains the ability to appoint judges to the SCT, and it has also come under criticism for its political application (Kamga and Fombad 2020).

There is observational evidence to suggest that partisan affiliation does in fact influence how Cameroonian citizens view legal institutions like the SCT. During Round 5 (2013) of the Afrobarometer, Cameroonians were asked two country-specific questions about Operation Sparrowhawk: whether they agreed that it has been an effective anticorruption tool, and whether they agreed that it is an instrument of repression against political opponents. All else held equal, regime partisans were far more likely to agree that Operation Sparrowhawk was effective, and to disagree that it is an instrument of repression, compared to opposition partisans and nonpartisans. In fact, partisanship was the only significant predictor of responses to these questions, outpacing both education and socioeconomic status (see Supplementary Material section H for details on this analysis).

### Design Strategy—Survey Experiment

There are challenges inherent to studying the effects of autocratic legalism. First, the available observational data only tells us what individuals think about a specific legal instrument, but not whether it actively legitimizes repression. Our question is whether autocratic legalism influences how individuals feel about acts of repression. Second, research in Cameroon—especially survey work—is costly and difficult. Parts of the country are very rural, where it is difficult to travel. Third, studying autocratic legalism in Cameroon is sensitive, and it can be difficult to recruit participants or have them speak openly. As noted below, these constraints drove decisions regarding research design but also limited the certainty of our results. Nonetheless, the constraints of working within difficult circumstances make the need for such work all the more imperative. As has been suggested elsewhere, findings that do not meet all the criteria for statistical significance can nonetheless be important, especially when the setting is new and there is little preexisting work (Gerber and Green 2012, pp. 61–63).

To assess the effects of autocratic legalism on perceptions of regime behavior, we embedded a survey experiment within a 1,200-respondent original survey conducted from January 20 to February 4, 2021.^4^ The survey was implemented face-to-face by a team of local interviewers in four of Cameroon’s ten regions—Centre, Littoral, Ouest, and Sud. The target population was adults of voting age in these four regions. Enumeration areas were selected to maximize variation in support for opposition parties within both rural and urban contexts, and concomitantly to balance the sample in terms of partisanship. To proxy opposition support, *départéments* were selected with current MPs from the ruling party (55 percent of the sample), MPs from the opposition (18 percent of the sample), and those with mixed representation (27 percent of the sample). Importantly, the “opposition strongholds” represent several different opposition parties. However, because of this specific sampling strategy, the data should not be considered nationally representative. In urban areas, interviewers stopped at every fifth house. In rural areas, where population densities were too low, interviewers stopped at every available house. Interviewers began at the same randomly chosen location within the enumeration area, walking in opposite directions. Interviewers surveyed Cameroonian citizens who were twenty years or older, which is the legal voting age in Cameroon. If the first person who responded was of voting age, they were chosen for the interview; if not, the interviewer asked if anyone of age was available for interview. One hundred thirty-eight households were double-sampled, such that two people in one house were interviewed. More details and a full list of enumeration areas are included in Supplementary Material section A.

Our experiment was included at the end of the survey and designed to capture the effects of exposure to autocratic legalism.^5^ Before reading the text of the experiment, interviewers told each respondent that although the scenario may sound familiar, it was hypothetical. The interviewer then read the following text:

*In the last few years, a minister has been critical of the government and announced the possibility of creating his own political party. Last year, the minister was arrested [****and tried in the new Special Criminal Tribunal****] and sentenced to 10 years in prison for allegedly embezzling 100,000,000 CFA. Do you think the minister’s sentence was appropriate?*

The treatment group was read the text about the SCT, while the control group was not. The difference in mean responses were then assessed across the full sample, but also according to partisan affiliation. To measure partisanship, the survey uses the same measure as the Afrobarometer, “Do you feel close to any particular political party?” followed by an open-ended response question regarding which specific party respondents feel close to. Interviewers were provided with a list of options to code from on the spot, including an “other” option, where they could write in a response, which was coded later. Respondents were only allowed to provide one option.

Concerns with construct validity and treatment effects drove this design. The scenario approximates political realities in Cameroon. The prosecution of controversial or oppositional elites is not unusual and the amount of alleged embezzlement is substantial and within the scope of the SCT. But the government has discretion, and such cases are not necessarily tried in the SCT. It was also important that the scenario allow for a degree of ambiguity regarding the minister’s guilt and that the sentence be appropriate to the alleged crime. The reference to the minister’s interest in creating his own political party leaves open the possibility that the accusation of embezzlement may be politically motivated. This makes it easier to discern the partisan effects of autocratic legalism. Likewise, by choosing a scenario that involves more subtle repression (a ten-year sentence would be reasonable), we are more likely to detect these effects. If we described a highly repressive scenario, such as the use of overt violence against ordinary citizens, the underlying act would likely draw stronger resistance regardless of whether it was justified by autocratic legalism or not (see Supplementary Material section I).

It is important to note that our survey experiment primes respondents differently based on whether they are familiar or not with the SCT. For those who are familiar, the treatment serves to remind them of the fact that it exists and makes it explicit that in this specific case it was invoked to try the accused minister rather than the regular judicial system. For those unfamiliar with the SCT, the prompt provides an entirely new piece of information that the prosecution of the minister was conducted under some specific judicial institution. Overall, two-thirds of our sample were familiar with the SCT, with opposition partisans more likely and nonpartisans slightly less likely to be familiar. We present results here for the full sample and include Supplementary Material section F that looks at the intersection of partisanship and awareness of the SCT.

Finally, given the sensitivity of the research question, only 50 percent of survey participants opted into the survey experiment. This raises concerns with the representativeness of our sample and our ability to discern treatment effects given sample sizes (especially in our subgroup analysis).^6^ As noted, our original sample frame was not representative and, as such, issues of strategic nonresponse are less concerning, especially since our experiment is well balanced across treatment and control (see table 1). In the Supplementary Material we compare respondents to nonrespondents across the entire sample, and within the critical group of nonpartisans (Supplementary Material section C). Respondents were more often male, educated, and aware of the SCT, but less likely to be nonpartisans. There were no differences in available measures of political trust or general political awareness. It is difficult to conclude how strategic nonresponse affects the representativeness of our results, but it is possible that our results for nonpartisans are slightly weaker than in reality.

**Table 1.** Covariate balance between treatment and control groups.

Covariate	(1)	(2)	(3)	(4)
	Control	Treatment	Difference of means	*p-v*alue
Gender	0.43	0.43	0.06	.874
	(302)	(281)	(0.04)	
Age	38.1	35.4	2.07*	.017
	(302)	(281)	(1.14)	
Education level	3.90	4.16	0.27	.080
	(302)	(280)	(0.15)	
Socioeconomic status	−0.03	0.17	0.20*	.016
	(288)	(274)	(0.09)	
Opposition partisan	0.25	0.27	0.02	.553
	(74)	(74)	(0.04)	
Regime partisan	0.33	0.27	0.06	.129
	(99)	(75)	(0.04)	
Nonpartisan	0.42	0.45	0.04	.377
	(125)	(125)	(0.04)	

*Note:* Mean values reported in columns 1–2 with sample sizes in parentheses. Differences of means reported in column 3 with standard errors in parentheses. *P-*values reported in column 4 (two-sample t-test).

*Represents differences with a *p*-value < 0.05.

## Results

As a preliminary test, we demonstrate that there are indeed partisan differences in perspectives of regime behavior, but also no overall treatment effect. Across both treatment and control groups, only 40.5 percent of opposition partisans believed that the minister’s sentence was appropriate, compared to 54.4 percent of nonpartisans and 65.5 percent of regime partisans. If approval of repression were uniform across these groups, we would question whether partisanship was an appropriate lens for understanding issues related to autocratic legalism or whether our scenario was an accurate depiction of repression for our sample. The appropriate responses to the scenario lend confidence to the general framework of the research design. However, unsurprisingly given our mixed expectations from different kinds of partisans, we find no average treatment effect across the full sample (see table 2). Therefore, there is limited evidence that autocratic legalism *consistently* impacts support for authoritarian behavior.

**Table 2.** Average treatment effects and average responses.

	Do you support the minister’s sentence?
Average treatment effect (standard error)	−0.04 (0.04)
	*p *=* *.378
Average response by treatment status (control, treatment)	0.56 (C), 0.52 (T)
Sample size	583

### Partisan Reactions to Autocratic Legalism

**Figure 1.**
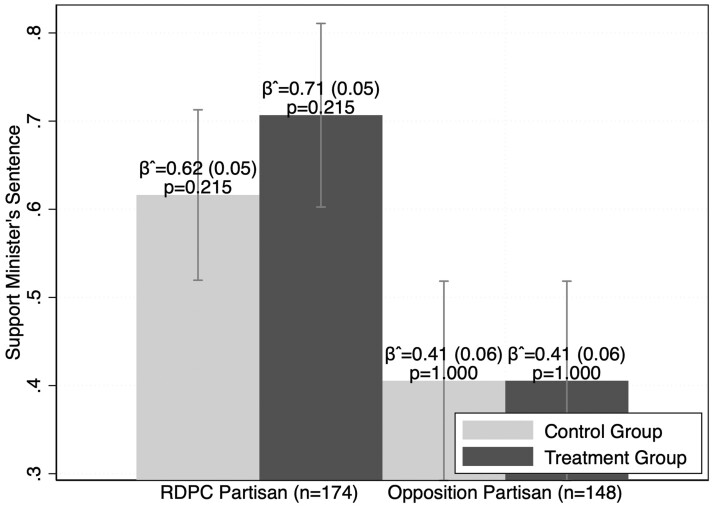
Responses by treatment status and partisanship. *Note*: Mean scores notated with 95 percent confidence intervals. Standard errors noted in parentheses.

Hypotheses 1 and 2 predict that reminding respondents of the SCT will increase support for autocratic behavior among regime partisans and decrease support among opposition partisans. Figure 1 contrasts the average response across treatment and control groups by partisan status. We surprisingly do not find strong support for either hypothesis in the data. Among regime partisans in our sample, there is a 9 percent jump in support for the minister’s sentence when the SCT is used to justify his trial, but this substantively large shift in opinion does not reach standard levels of statistical significance.^7^ Opposition partisans, on the other hand, have no reaction at all to autocratic legalism, consistent with the notion that their preexisting opposition to the regime makes it difficult to detect effects.

Though we hypothesized that partisans would react most strongly to appeals of autocratic legalism, it turns out that they have little reaction, particularly opposition partisans, who exhibit no treatment effect at all. Given issues with the sample size, there is a possibility that we have failed to detect a positive effect among ruling-party partisans, but if so, the effect would appear to be small. Although the partisans in our sample do not react to autocratic legalism, our findings do not contradict the core logic of the meaning of partisanship in electoral autocracies. Opposition partisans have a much lower tolerance for repressive acts than do ruling-party partisans. Their baseline acceptance of political repression is already so low and closely held that invoking autocratic institutions to justify the repression does little to change their preexisting beliefs. Inversely, ruling-party partisans are so trusting of the regime that their acceptance of repressive acts is not really affected by legal justifications.

### Nonpartisan Reactions to Autocratic Legalism

**Figure 2.**
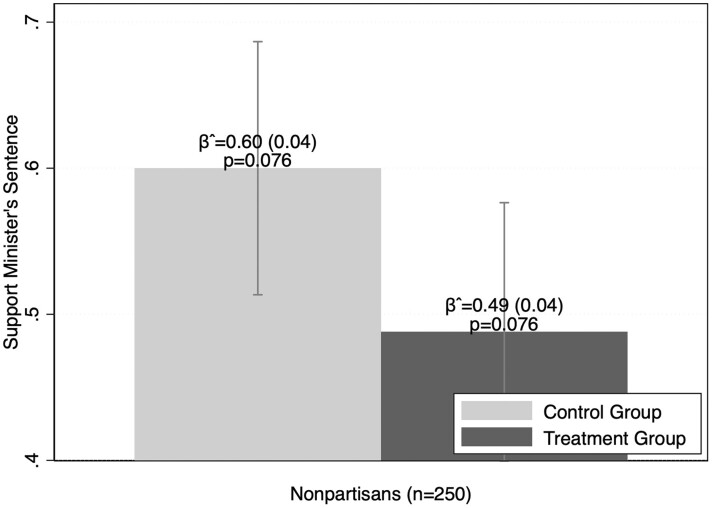
Responses by treatment status among nonpartisans. *Note*: Mean scores notated with 95 percent confidence intervals. Standard errors noted in parentheses.

Given how little we know about nonpartisans in electoral autocracies, we do not engage in hypothesis testing of expectations about this group of citizens. Instead, we present post hoc exploratory data based on two different interpretations of the meaning of nonpartisanship under electoral authoritarianism. One proposes that if nonpartisans are truly apolitical, they likely will have no reaction to legal justifications for repression. In fact, our data show that nonpartisans have a substantial negative reaction to the treatment (albeit only bordering on statistical significance). As shown in figure 2, nonpartisans reduce their support for the arrest of the allegedly corrupt minister by 11 percentage points when exposed to the legal justification.

A second possibility is that nonpartisans are not apolitical, but instead “closeted partisans.” Although they do not actively align with one political party or another, they should exhibit political preferences that align with either ruling-party partisans or opposition partisans. To classify nonpartisans into “ruling-party leaners” and “opposition leaners,” we split the group of nonpartisans into those who say they trust the president “a lot” or “somewhat” (ruling-party leaners) from those who trust him “a little” or “not at all” (opposition leaners). Considering the results for hypotheses 1 and 2, if nonpartisans are “closeted partisans,” then in fact, once split into their respective groups, they should have no reaction to the treatment. However, we *would* expect ruling-party leaners to be more likely to accept repression (irrespective of the mention of the SCT) and opposition leaners to be less likely to accept repression.

**Figure 3.**
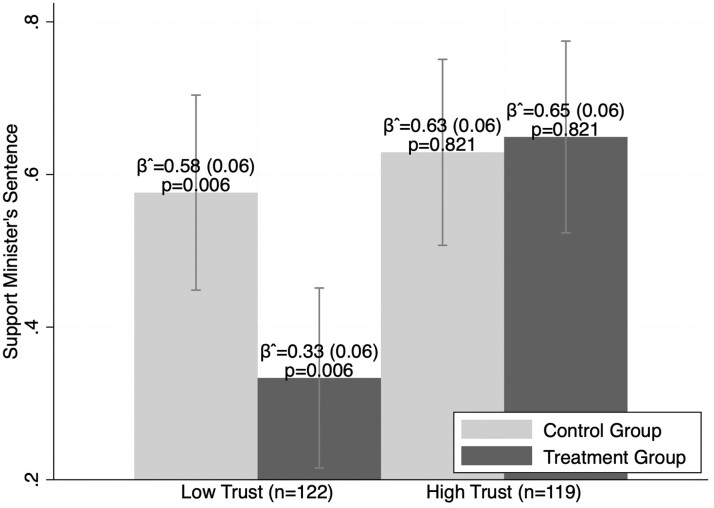
Responses by treatment status among nonpartisans with low levels of trust in the president and high levels of trust. *Note*: Mean scores notated with 95 percent confidence intervals. Standard errors noted in parentheses.

The data from the survey experiment among low-trust and high-trust nonpartisans are mixed. As reported in figure 3, nonpartisans in our sample who trust the president (ruling-party leaners) look almost identical to the ruling-party partisans in our sample. Their support for the minister’s sentence (63.9 percent across treatment and control) is nearly identical to that of ruling-party partisans (65.5 percent across treatment and control). Likewise, the invocation of autocratic legalism does not change their support of the repression. By contrast, nonpartisans with low levels of trust in the president (opposition leaners) are strongly affected by the treatment. Their support for the minister’s sentence nearly halves, dropping from 58 percent in the control group (a figure similar to ruling-party partisans) to 33 percent in the treatment, which is even lower than opposition support (46 percent across treatment and control).^8^ Notably, these results are statistically significant, and as explained in Supplementary Material section G, adequately powered to detect such a large effect.

## Post Hoc Interpretation of “Closeted Opposition Partisans”

Although we did not test hypotheses about nonpartisans, we did explore their reactions to autocratic legalism. In this post hoc exploration, we found that nonpartisans—specifically, nonpartisans who don’t trust the president—react very strongly, and negatively, to autocratic legalism. We present some potential interpretations of the meaning of partisanship based on these findings; however, further data must be collected to test these interpretations, both inside Cameroon as well as in other electoral autocracies. On the one hand, it appears that some nonpartisans are in fact “closeted ruling-party partisans.” Though these nonpartisans do not feel close to the ruling party, they trust the president and accept acts of repression at levels almost identical to those of ruling-party partisans. Also, like ruling-party partisans, appeals to autocratic legalism do little to change their acceptance of these acts of repression. On the other hand, we do not find evidence for an inverse group of “closeted opposition partisans.” Nonpartisans who do not trust the president do not act like opposition partisans. They approve of repression against opposition leaders at levels that are similar to ruling-party partisans (and ruling-party leaners), but when the SCT is invoked to justify this repression, suddenly they look a lot more like opposition partisans—their support for repression plummets.

We tentatively propose that these antiregime nonpartisans are potentially a group unique to electoral autocracies. In cases like Cameroon, it is possible that some nonpartisans are actually “anti-establishmentarians.” They may distrust the regime and disapprove of its policies as much as opposition partisans, but unlike opposition partisans, these nonpartisans also distrust opposition parties and find them to be just as reprehensible as the regime. Opposition parties in electoral autocracies tend not to be very popular. Since they cannot win elections, they have little to offer in terms of policy promises or access to patronage, and partially as a result, they tend to be much more ethnic or regional in nature (Stroh 2010; Letsa 2017, 2019). Thus, citizens from outside these “opposition regions” may be naturally mistrustful of parties that appear socially distinct from themselves. Indeed, the Round 6 Afrobarometer sample of electoral autocracies asked respondents how much they trusted twelve different political institutions or actors, and nonpartisans collectively ranked “opposition parties” dead last—even lower than the ruling party, the police, and the tax department.

Thus, unlike nonpartisans in democratic regimes who may be true “closeted partisans,” we propose that some proportion of nonpartisans in electoral autocracies may be politically aware, but not ideologically similar to either ruling-party or opposition partisans. These kinds of nonpartisans are hyperoppositional to the entire political system. If this is the case, it would explain why we find that they hold ambivalent views about repression against opposition actors but react negatively to the invocation of legal justifications for said repression, because such institutions represent the illegitimacy of the autocratic regime.

## Conclusion

This article has used the case of Cameroon to explore how individuals adjust their opinions regarding repression based on their exposure to autocratic legalism. In general, the findings from our survey experiment demonstrate that autocratic legalism is not particularly effective. It appears to have little effect among citizens who already have strong partisan commitments and produces a negative response among the nonpartisans in our sample, especially those with higher levels of regime distrust. These findings, while tentative, offer difficult to gather yet novel insights into autocratic legalism, demonstrate the key role of partisanship, and offer new insights into nonpartisanship in authoritarian regimes.

The findings raise critical questions about the aims of autocratic legalism as well as the meaning of partisanship in electoral authoritarian regimes. The overall null effects of exposure to autocratic legalism suggest that either such innovations often fail to elicit the desired domestic response, or they are created to satisfy international or intraregime audiences. On the other hand, the large negative effect found among nonpartisans is congruent with other work on the effects of propaganda in polarized societies. Attempts at domestic legitimation can backfire and sow seeds of further distrust among certain groups (Peisakhin and Rozenas 2018; Graham and Svolik 2020; Chapman 2021). This article pushes that insight further into the role of autocratic legalism and a recasting of the meaning of nonpartisanship. Nonpartisanship is a key social category that is underexplored in electoral authoritarian regimes, and, yet, in Cameroon it categorizes most citizens. Our findings demonstrate that nonpartisans are not swing voters or clearly closeted partisans. Rather, a significant proportion are more likely anti-establishmentarian—distrustful of most political parties and institutions. Future work should consider how authoritarian strategies of political communication and institutional innovation intersect with more complex notions of what the electorate looks like and what nonpartisanship means.

## Supplementary Material

nfad051_Supplementary_Data

## Data Availability

Replication data and documentation are available at https://doi.org/10.7910/DVN/GM8DMG.

## Footnotes

1 Cameroon, Tanzania, Togo, Uganda, and Zimbabwe.

2 It is not clear whether this indicates that nonpartisans are split between leaning toward the regime and opposition, or that they simply hold more neutral positions because they are generally apolitical.

3 This has been established in several human rights reports between 2004 and 2011. See also the excellent overview in https://www.jeuneafrique.com/186293/politique/paul-biya-accus-d-instrumentaliser-epervier/.

4 The overall response rate for the survey was 62.2 percent. The response rate was calculated as the number of participants who agreed to participate (1,200) divided by the total number of people who were asked to participate (1,930). If no one answered the door at a residence, it was not included in the denominator for the response rate.

5 We were advised by the survey firm that this experiment may be perceived as particularly sensitive in the current political climate in Cameroon, so we moved the question to the end of the survey and allow respondents to “opt in” to the questions. Therefore, each respondent was read this prompt at the end of the survey: “For the final questions of the survey, I want to ask you your opinion about a few things that some people might find politically sensitive. Just like the rest of the survey, your responses will be completely anonymous. But before we begin, I want to remind you that you are free to withdraw from this survey and need not answer any questions that you do not want to answer.”

6 The issue of nonresponse made it difficult to calculate sample sizes, as did the fact that there is no preexisting reference treatment effect. We present an ex ante power analysis in Supplementary Material section D, which demonstrates that we are adequately powered to detect medium-sized effects in our subgroup analysis (using Cohen’s d as a reference). We are therefore confident that our null results are not driven by a lack of power. Likewise, we report an ex post power analysis to show what sample size we would have needed to achieve 80 percent power with the observed treatment effects. It suggests that a lack of statistical power does not explain our null results. Our significant results are at times underpowered, and we therefore might be exaggerating the treatment effect or falsely rejecting the null hypothesis.

7 It is possible that we have failed to detect a statistically significantly effect because the subgroup of ruling-party partisans is underpowered; we would have needed a sample size of 900 ruling-party partisans to reach statistical significance on a treatment effect of 0.09.

8 Significantly, given our emphasis on the importance of partisanship as a framework for understanding support for autocratic legalism, trust in the president alone does not produce any treatment effects (see Supplementary Material section G).
