# Supplementary material for: Autocratic Legalism, Partisanship, and Popular Legitimation in Authoritarian Cameroon
Source: Public Opin Q. 2023 Dec 12;87(4):935–55. doi: 10.1093/poq/nfad051 (PMC10748577; doi:10.1093/poq/nfad051)
Supplement: nfad051_Supplementary_Data [file nfad051_supplementary_data.pdf]

Supplementary Material for

Autocratic Legalism, Partisanship, and Popular Legitimation in Authoritarian Cameroon

NATALIE WENZELL LETSA: Assistant Professor, Department of International and Area Studies, University of Oklahoma, Norman, OK, US.

YONATAN L. MORSE: Associate Professor, Department of Political Science, University of Connecticut, Storrs, CT, US.

SUPPLEMENTARY INFORMATION

Section A: Enumeration Areas

Section B: Descriptive Statistics

Section C: Comparing Respondents to Nonrespondents

Section D: Power Analysis

Section E: Randomization Inference

Section F: Responses by Level of Awareness of Special Criminal Tribunal

Section G: Survey Experiment Results for Low Trust and High Trust sub-Groups

Section H: Analysis of Afrobarometer Data on 'Operation Sparrowhawk'

Section I: Alternative Scenario (2014 Anti-Terror Law)

Section A: Enumeration Areas

The survey was conducted by the Cible Groupe. A total of 1,200 respondents were interviewed from January 20 – February 4, 2021. Table A1 presents every district sampled in the survey. These areas were sampled on two characteristics. First, whether the *département* historically votes for an opposition political party, the ruling party, or "swings" between the two. Second, variation in urban and rural EAs were created for each of these three subgroups of *départements*.

In urban areas, enumerators stopped at every 5th house. In rural areas, where population densities were too low, enumerators stopped at every available house. Enumerators began at the same randomly chosen location within the enumeration area, walking in opposite directions. Enumerators interviewed Cameroonian citizens who were twenty years or older; the legal voting age in Cameroon is 20. 138 households were double-sampled, such that two people in one house were interviewed.

Table A1 Enumeration Areas

Region	Département	Arrondissement
Centre	Lékié Ouest	Evodoula
	Mbam et Inoubou	Bokito, Kon-Yambetta, Makenéné, Ndikinimeki
	Mfoundi	Yaoundé I, Yaoundé II
	Nyong et Kellé	Eséka, Messondo, Ngog-Mapubi
Littoral	Nkam	Nkondjock, Nord Makombe, Yabassi
	Sanaga Maritime	Edéa II, Ngwei
	Wouri East	Douala III
	Wouri Centre	Douala I
	Wouri Sud	Douala II
South	Dja et Lobo	Sangmelima, Zoétélé
	Mvila	Ebolowa I
	Océan	Lokoundjé
West	Haut Nkam	Bafang
	Haut Plateaux	Baham, Bamendjou, Bangou
	Mifi	Bafoussam
	Ndé	Bazou
	Noun Centre	Foumban, Malentouen, Massangan

Section B: Descriptive Statistics

Table B1 Descriptive Statistics

Variable	**N**	**Mean**	**SD**	**Min**	**Max**
Feel Close to Party	1,177	0.50	0.50	0	1
Nonpartisan	1,164	0.50	0.50	0	1
RDPC Partisan	1,164	0.28	0.45	0	1
Opposition Partisan	1,164	0.21	0.41	0	1
Support Minister's Trial	583	0.54	0.49	0	1
Treatment	583	0.48	0.50	0	1
Heard of the Special Criminal Tribunal	1,172	0.94	0.86	0	2
Trust in Paul Biya	1,125	1.67	1.20	0	3
Age	1,200	37.18	14.28	20	95
Gender	1,200	0.50	0.50	0	1
Education	1,193	3.76	1.88	0	8
Urban	1,200	0.76	0.89	0	2
SES Factor	1,141	0.00	1	-3.04	2.53
Freedom Factor	1,122	0.00	1	-3.04	1.12
News Factor	1,129	0.00	1	-1.70	2.37

Note: Education was rated on a 0 to 7 scale. The SES Factor is the product of a factor analysis that asked respondents to note their assets (radio, television, car, motorcycle, mobile phone, computer, refrigerator, bicycle, passport, bank account). The survey included questions that asked respondents to mark their level of trust in the president and the party along a four-point scale (Not at all, Just a little, Somewhat, A lot). The News Factor is the product of a factor analysis that asked respondents to mark how frequently they consumed radio, television, newspapers, and social media (Never, Less than once a month, Several times a month, Several times a week, Everyday). The Freedom Factor is the product of a factor analysis that asked respondents to rate their freedom to say what you think, join a political organization, and vote (Not at all free, Not very free, Somewhat free, Completely free).

Section C: Comparing Respondents to Nonrespondents

Strategic nonresponse can induce bias into survey results when nonrespondents differ in significant ways from respondents along critical characteristics. Our survey was not meant to be nationally representative, and we achieved effective randomization across treatment and control. Table C1 reports key demographic data, information about partisanship, and measures of political awareness and trust. Table C2 reports similar information just within the subgroup of identified nonpartisans.

Table C1 Comparing Respondents to Nonrespondents (Full Sample)

	Respondents	Nonrespondents	*P*-Value
% Female	0.43	0.56	0.000
	(0.02)	(0.02)	
Age	36.8	37.5	0.356
	(0.57)	(0.59)	
Education	4.0	3.5	0.000
	(0.08)	(0.08)	
Socioeconomic Status (SES Factor)	0.07	-0.07	0.020
	(0.04)	(0.04)	
Urban	0.77	0.77	0.948
	(0.04)	(0.04)	
% Feel Close to Party	56.2%	44.7%	0.000
	(0.02)	(0.02)	
% CPDM Partisan	30.4%	26.7%	0.159
	(0.02)	(0.02)	
% Opposition Partisan	25.9%	17.1%	0.000
	(0.02)	(0.02)	
% Non-Partisan	43.7%	56.2%	0.000
	(0.02)	(0.02)	
Heard of SCT	1.08	0.82	0.000
	(0.04)	(0.03)	
Trust Paul Biya	1.64	1.70	0.410
	(0.05)	(0.05)	
Trust CPDM	1.30	1.41	0.051
	(0.05)	(0.05)	
News Factor	0.04	-0.04	0.214
	(0.04)	(0.04)	
Freedom Factor	-0.01	0.02	0.606
	(0.04)	(0.04)	

Note: Results of two-sample t-test. Education was rated on a 0 to 7 scale. The SES Factor is the product of a factor analysis that asked respondents to note their assets (radio, television, car, motorcycle, mobile phone, computer, refrigerator, bicycle, passport, bank account). The survey included questions that asked respondents to mark their level of trust in the president and the party along a four-point scale (Not at all, Just a little, Somewhat, A lot). The News Factor is the product of a factor analysis that asked respondents to mark how frequently they consumed radio, television, newspapers, and social media (Never, Less than once a month, Several times a month, Several times a week, Everyday). The Freedom Factor is the product of a factor analysis that asked respondents to rate their freedom to say what you think, join a political organization, and vote (Not at all free, Not very free, Somewhat free, Completely free).

Table C2 Comparing Respondents to Nonrespondents (Nonpartisans Only)

	Nonpartisan Respondents	Nonpartisan Nonrespondents	*P*-Value
% Female	0.51	0.60	0.021
	(0.03)	(0.03)	
Age	33.8	35.22	0.202
	(11.8)	(14.4)	
Education	3.53	3.98	0.003
	(0.10)	(0.11)	
Socioeconomic Status (SES Factor)	0.10	0.06	0.593
	(0.06)	(0.05)	
Urban	0.81	0.82	0.956
	(0.04)	(0.06)	
Heard of SCT	0.95	0.76	0.009
	(0.05)	(0.04)	
Trust Paul Biya	1.53	1.63	0.336
	(0.08)	(0.06)	
Trust CPDM	1.13	1.30	0.108
	(0.07)	(0.06)	
News Factor	0.03	-0.04	0.368
	(0.06)	(0.06)	
Freedom Factor	-0.15	-0.16	0.878
	(0.06)	(0.06)	

Note: Results of two-sample t-test. Education was rated on a 0 to 7 scale. The SES Factor is the product of a factor analysis that asked respondents to note their assets (radio, television, car, motorcycle, mobile phone, computer, refrigerator, bicycle, passport, bank account). The survey included questions that asked respondents to mark their level of trust in the president and the party along a four-point scale (Not at all, Just a little, Somewhat, A lot). The News Factor is the product of a factor analysis that asked respondents to mark how frequently they consumed radio, television, newspapers, and social media (Never, Less than once a month, Several times a month, Several times a week, Everyday). The Freedom Factor is the product of a factor analysis that asked respondents to rate their freedom to say what you think, join a political organization, and vote (Not at all free, Not very free, Somewhat free, Completely free).

Section D: Power Analysis

Statistical power refers to the sensitivity of a hypothesis test to Type II error, or the probability of finding an effect if there is an effect to be found. It is sensitive to sample size, expected treatment effects, and the strength of treatment. Without guidance from pre-existing research, we use Cohen's d as a benchmark. Using the pooled standard deviation of 0.5, a small effect would be equivalent to a 0.1 difference in means and a medium effect to a 0.25 difference in means. An *ex-ante* power analysis was conducted using State 17.0, which runs multiple iterations to calculate sample size based on the desired treatment effects. In addition, we conducted an *ex-post* power analysis that calculates what the sample size would have needed to be in order to detect the treatment effect that we actually observe. The results for the full sample and the partisan subgroups are plotted in Figure D1. The results for sub-groups of nonpartisans based on levels of trust are reported in Figure D2.

	(a) Detectable Effect Size with Current Sample Size (ex-ante power)	(b) Current Power and Sample Size Needed for Estimated Effect Size (ex-post power)
Full Sample	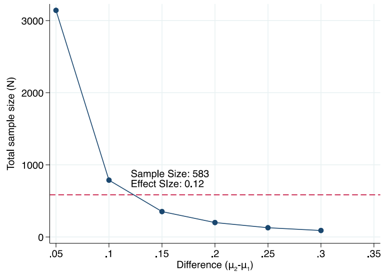	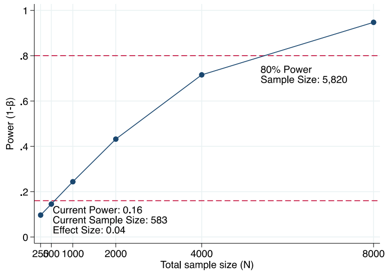
Non-Partisans	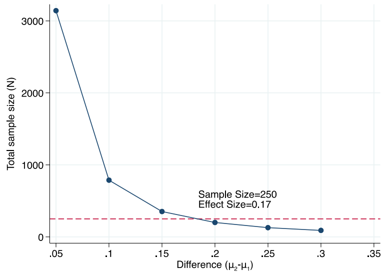	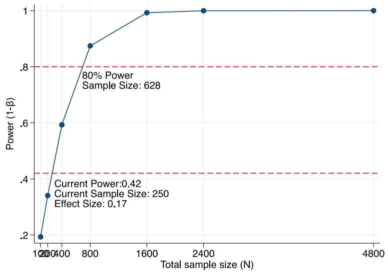
Regime Partisans	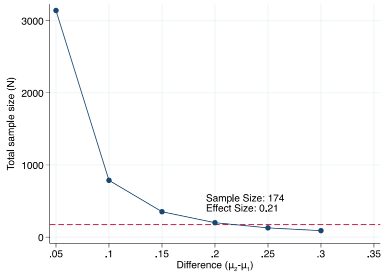	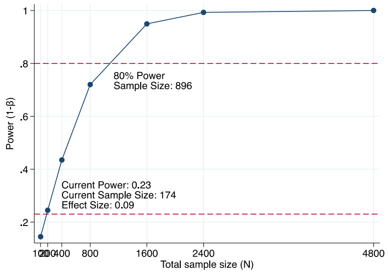
Opposition Partisans	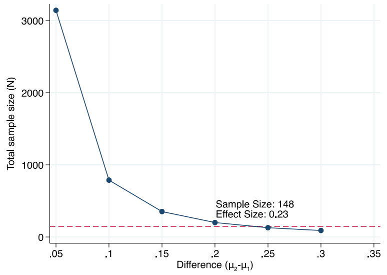	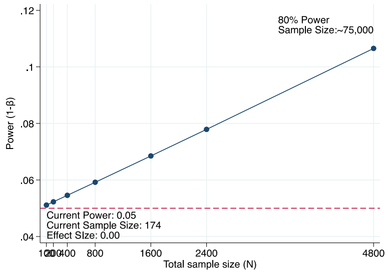

Figure D1 Ex-Ante and Ex-Post Power Analysis (Full Sample and Partisan Sub Groups). *Note:* The dotted red line in column (a) plots the size of our sample and the detectable treatment effect at that sample size. The bottom dotted red line in column (b) reports the actual statistical power we achieve given the sample size and treatment effect, while the top dotted line plots what the sample size would have needed to be to achieve 80% statistical power.

<table>
<tr><th></th><th>(a) Detectable Effect Size with Current Sample Size (ex ante)</th><th>(b) Current Power and Sample Size Needed for Estimated Effect Size (ex post)</th></tr>
<tr><td>Non-Partisan / High Trust</td><td>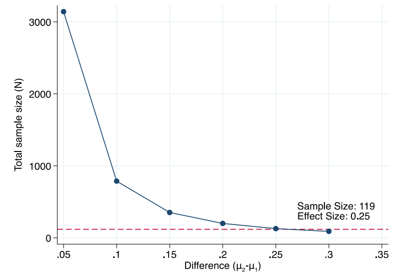
</td><td>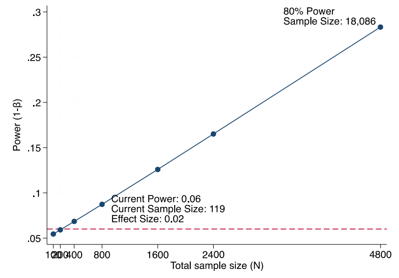
</td></tr>
<tr><td>Non-Partisan / Do Not Low Trust</td><td>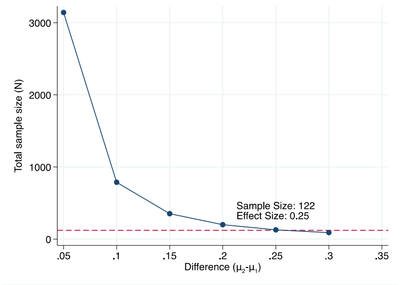
</td><td>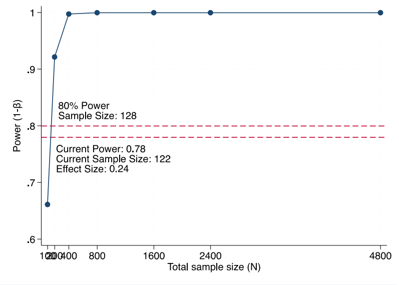
</td></tr>
</table>

Figure D2 Ex-Ante and Ex-Post Power Analysis (Nonpartisan Sub-Groups). *Note:* The dotted red line in column (a) plots the size of our sample and the detectable treatment effect at that sample size. The bottom dotted red line in column (b) reports the actual statistical power we achieve given the sample size and treatment effect, while the top dotted line plots what the sample size would have needed to be to achieve 80% statistical power.

Section E: Randomization Inference

With small sample sizes it is useful to include Randomization Inference p-values. Randomization inference compares the actual estimated treatment effects ($\hat{\beta}$) to a reference distribution based on a large number of simulations (1,000). This provides an assessment of how likely it is to obtain an effect more extreme than the one reported.

We run regression models of our survey outcomes and treatment and calculate RI p-values using Stata's *ritest* commands. An original regression model is estimated and saved as a point estimate of the treatment indicator. This is repeated 1,000 times and plotted as an estimate of the treatment effect. We then calculate the RI p-value, which is the proportion of times the simulated treatment was more extreme in a two-tail test than the actual estimated treatment effect reported in the main text. Both the original and imputed P-Values are reported in Figure E1.

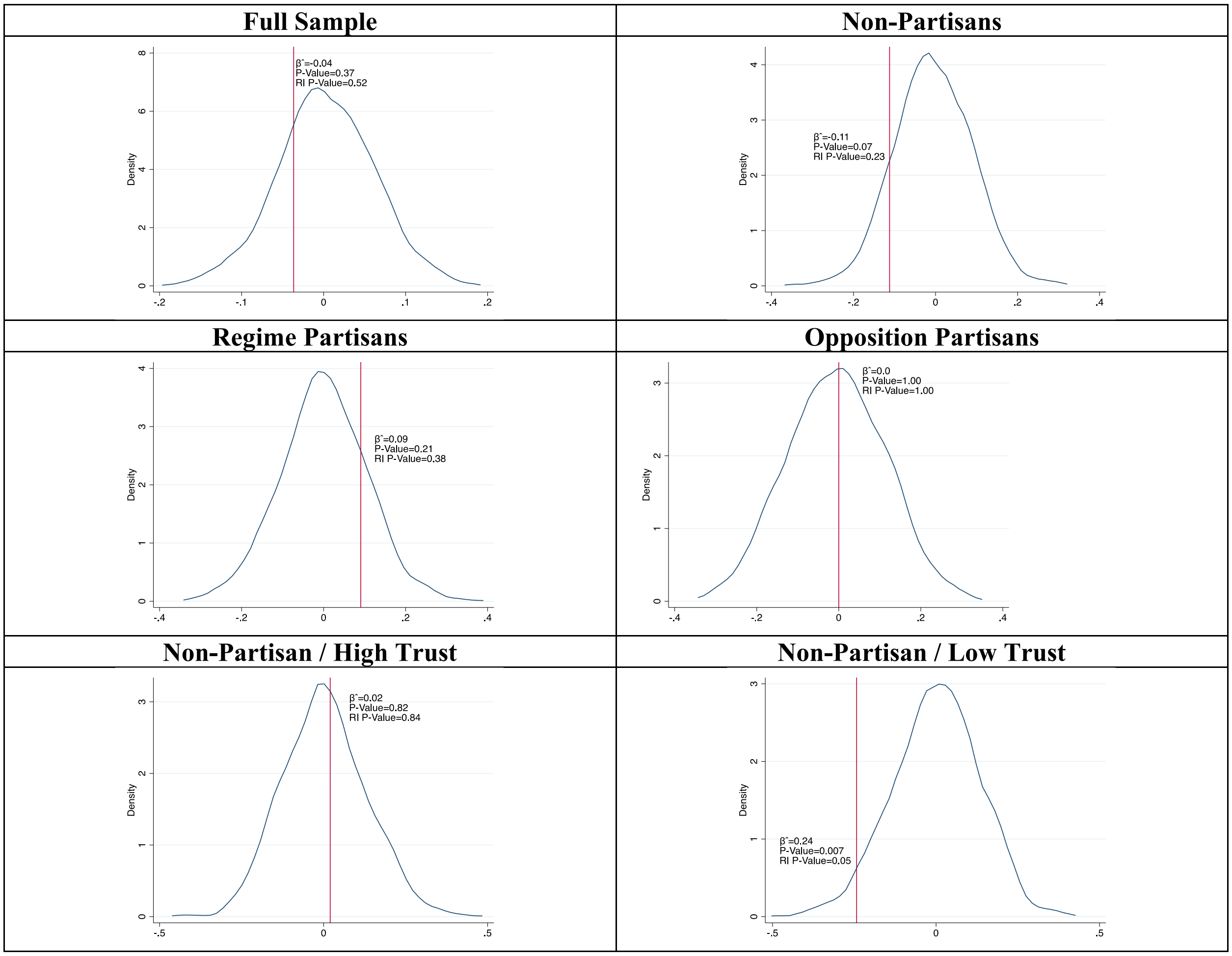

Figure E1 Randomization Inference P-Values.

Section F: Survey Experiment Results Based on Pre-Existing Knowledge of Special Criminal Tribunal

Figure F1 shows that when the sample is restricted to those who have heard of the Special Criminal Tribunal, the effect of autocratic legalism is still negative and significant among nonpartisans. There is still no treatment effect among opposition or RDPC partisans. On the other hand, when the sample is restricted to those who have not heard of the Special Criminal Tribunal, there is still a negative response among nonpartisans but it is not statistically significant. Note however that sample sizes are much smaller with this restriction. Among nonpartisans, 150 have heard of the tribunal and 100 have not. Among RDPC partisans 121 have heard of the tribunal and 53 have not. Among opposition partisans 114 have heard of the tribunal and 34 have not.

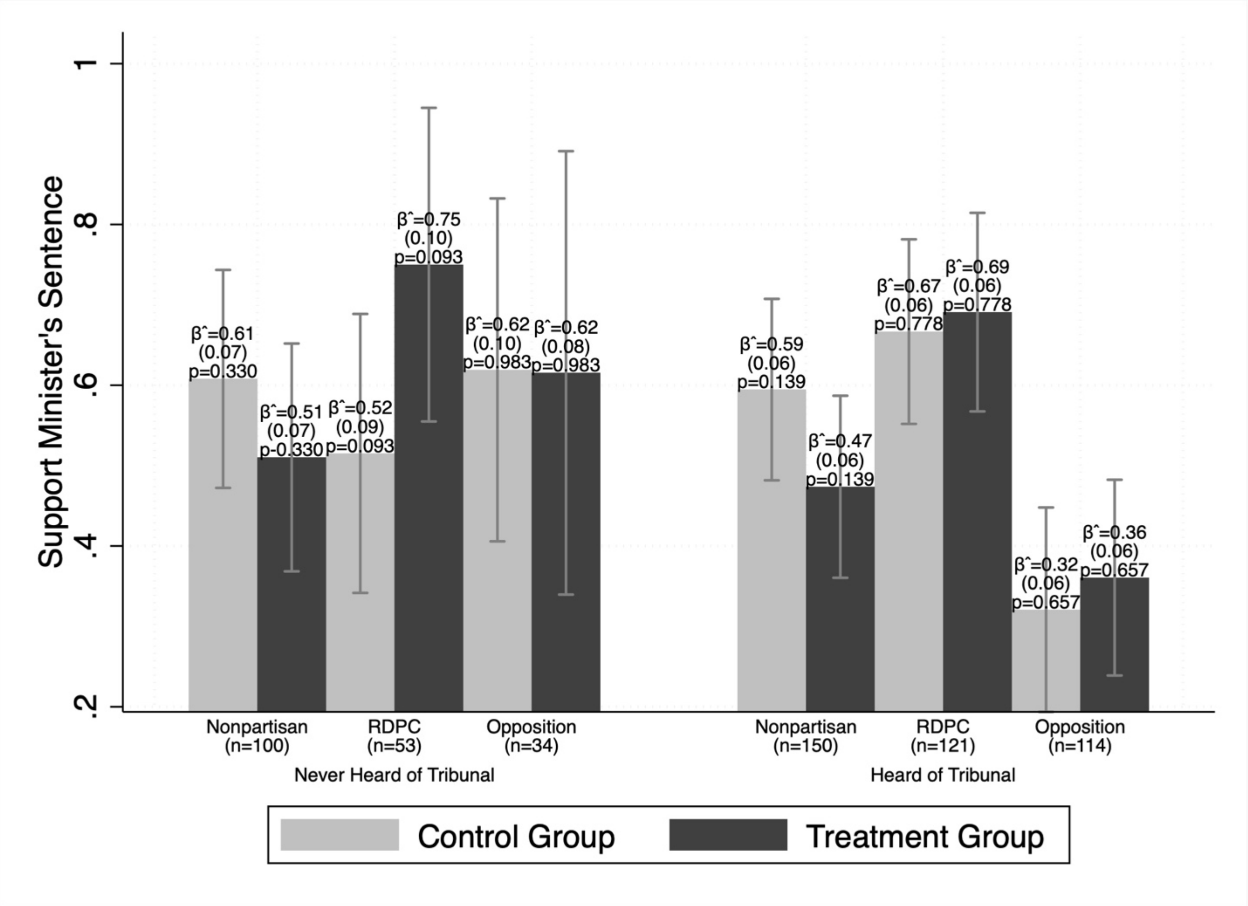

Figure F1 Responses by Treatment Status amongst respondents based on awareness of SCT. *Note*: Mean scores notated with 95% confidence intervals. Standard errors noted in parentheses.

Section G: Survey Experiment Results for Low Trust and High Trust sub-Groups

Figure 4 reveals that when nonpartisans are broken down between those who have high levels of trust in the president and those who have low levels of trust, the effect of autocratic legalism has an even more pronounced negative effect amongst the low-trust group. For high trust nonpartisans, there appears to be no treatment effect. This finding might suggest that trust in the president alone may be a sufficient lens through which to understand the effects of autocratic legalism; that perhaps partisanship is not as important as trust in the regime. However, when we split the sample between low-trust and high-trust respondents, as seen in Figure G1, there appears to be no treatment affect amongst either group. We believe that partisanship is a better framework for understanding the legitimating effect of autocratic legalism because it measures a level of political engagement through the category of nonpartisanship, which trust does not. By looking at trust alone, we would think autocratic legalism had no legitimating affect at all, when in fact it does have a large effect amongst nonpartisans, who are politically disengaged, but inclined to be distrustful of the regime.

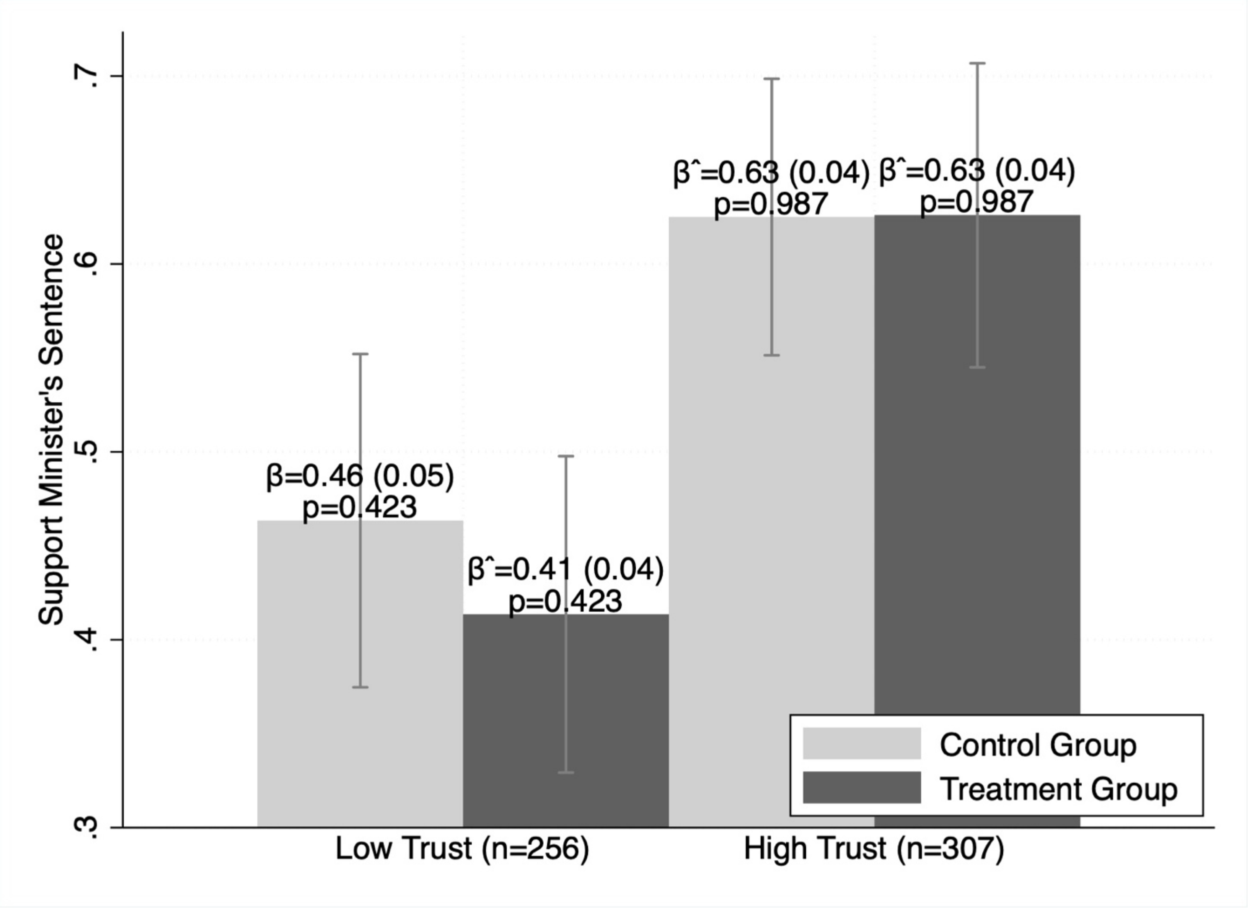

Figure G1 Responses by Treatment Status amongst respondents with low levels of trust in the president and high levels of trust. *Note*: Mean scores notated with 95% confidence intervals. Standard errors size noted in parentheses.

Section H: Analysis of Afrobarometer Data on 'Operation Sparrowhawk'

Round 5 (2013) of the Afrobaromter asked two country-specific questions in Cameroon about Operation Sparrowhawk:

Do you agree or disagree with the following statements about Operation Sparrowhawk?

1. *The fight against corruption and the embezzlement of public funds by civil servants, Operation Sparrowhawk, has been effective.*
2. *The fight against corruption and embezzlement of public funds by officials, Operation Sparrowhawk, is an instrument of repression against adversaries who have political ambitions.*

Respondents could choose from a five-point scale, ranging from "totally disagree" to "completely agree." For the first question, 59.6 percent of the full sample agreed that the Operation Sparrowhawk has been effective, compared to 32.8 percent who disagreed. Inversely, for the second question, 41.8 percent agreed that it was an instrument of repression, while 43.9 percent disagreed. Interestingly, although responses to the questions were (negatively) correlated, a substantial group of respondents (21.8 percent) believed that Operation Sparrowhawk has been effective at combatting corruption while also acting as a tool for repression.

Using these two questions as our dependent variables, we run simple OLS regressions with partisanship, gender, age, urban/rural status, education and socioeconomic status as predictors. To measure partisanship, the survey asked, "Do you feel close to any particular political party?" Those who responded no were coded as nonpartisans (58.3 percent of the full sample). Those who responded 'yes' were then asked, "Which party do you feel close to?" Respondents who reported the RDPC were coded as regime partisans (31.8 percent), while those who reported an opposition party were coded as opposition partisans (9.9 percent). Education was reported on a ten-point scale from "no formal education" to "graduate degree." Finally, in order to measure socioeconomic status, we created a factor variable out of six questions: whether or not the respondent owned a

radio, television, car or motorcycle, the quality of their water source and latrine, and finally, the extent to which they had gone without food to eat in the past twelve months. We rescaled the variable to range from zero to ten. Standard errors in each regression were clustered at the level of the enumeration area.

The results of the regressions are reported in Figure H1, where the first graph plots the coefficients for these predictors of the first question—the effectiveness of Operation Sparrowhawk—while the second graph plots them for the second question—whether or not Operation Sparrowhawk is a tool of political repression. The effect of partisanship is clearly large for both questions. All else held equal, the difference between a regime partisan and anyone else results in a 0.62-point increase on the 5-point scale. In comparison, if we look at the full range of educational outcomes—someone with no education compared to someone with a post-graduate degree, there is a 0.89-point decrease in support for Operation Sparrowhawk.

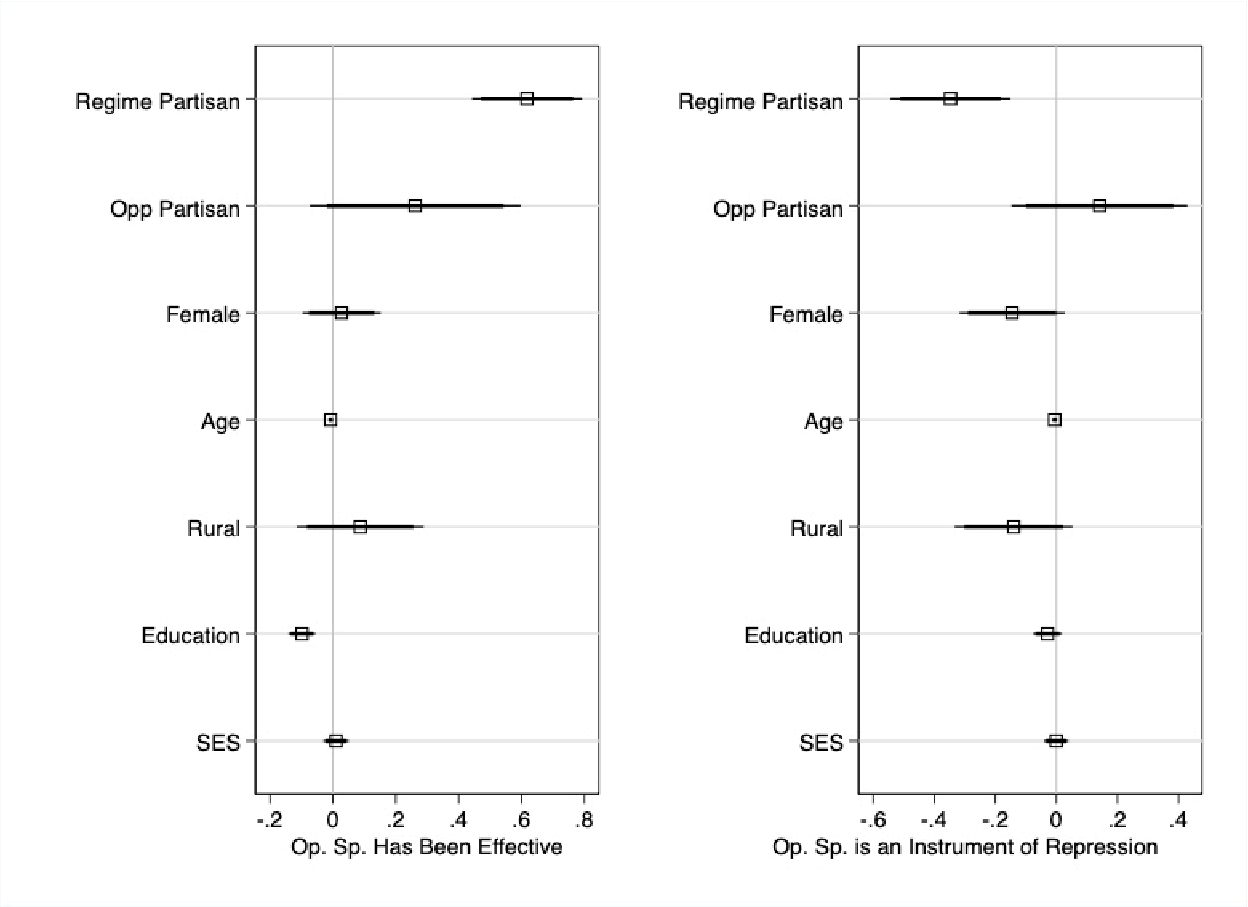

Figure H1 Coefficient plots for 1) support for Operation Sparrowhawk and 2) belief that Operation Sparrowhawk is an instrument of repression. *Note:* N = 929. Coefficients displayed with 90% and 95% confidence intervals. Standard errors clustered at the enumeration area.

The magnitude of the effect is even larger for the second question, where controlling for all of these demographic factors, partisanship is in fact the only statistically significant predictor of whether or not someone thinks that Operation Sparrowhawk is a tool of political repression. These results indicate that if there is any predictable framework for understanding beliefs about autocratic legalism, partisanship is at least more reliable than traditional predictors of public opinion, such as education or socioeconomic status. Ruling party partisans appear to view these autocratic tools very differently than do their nonpartisan or opposition counterparts. However, due to the nature of the observational data, the analysis cannot tell us whether or not autocratic legalism actually legitimizes acts of repression—either in the eyes of all citizens, or specifically in

the eyes of regime partisans. Thus, the following section presents data from a survey experiment that can provide such a causal framework for answering this question.

Section I: Alternative Scenario (High Repression)

Our survey also included an alternative scenario involving the detention of opposition protestors and the impact of an anti-terror law passed in 2014. The prompt read as follows with the treatment noted in brackets:
It was recently reported that several peaceful protestors in Yaoundé were arrested by security forces and detained in Kondengui Central prison [under the anti-terrorism law that passed in 2014]. The government declared that the protestors represented a threat to national security. The protestors were released with no charges after 14 days. Do you think that the arrest of the protestors was well-justified?

As with the Special Criminal Tribunal, our sample of respondents was slightly more aware of the 2014 Anti-Terror Law. There was no overall treatment effect (Table I1) or effects by partisanship Figures I1 and I2). In addition, there was across the board comparatively less support for the underlying repressive act.

Table I1 Average Treatment Effects and Average Responses

	Do you support the arrest of the protestors?
Average Treatment Effect (ATE)	-0.02 (0.04)<br>*p*=0.651
Average Response by Treatment Status (Control, Treatment)	0.43 (C), 0.42 (T)
Sample Size	611

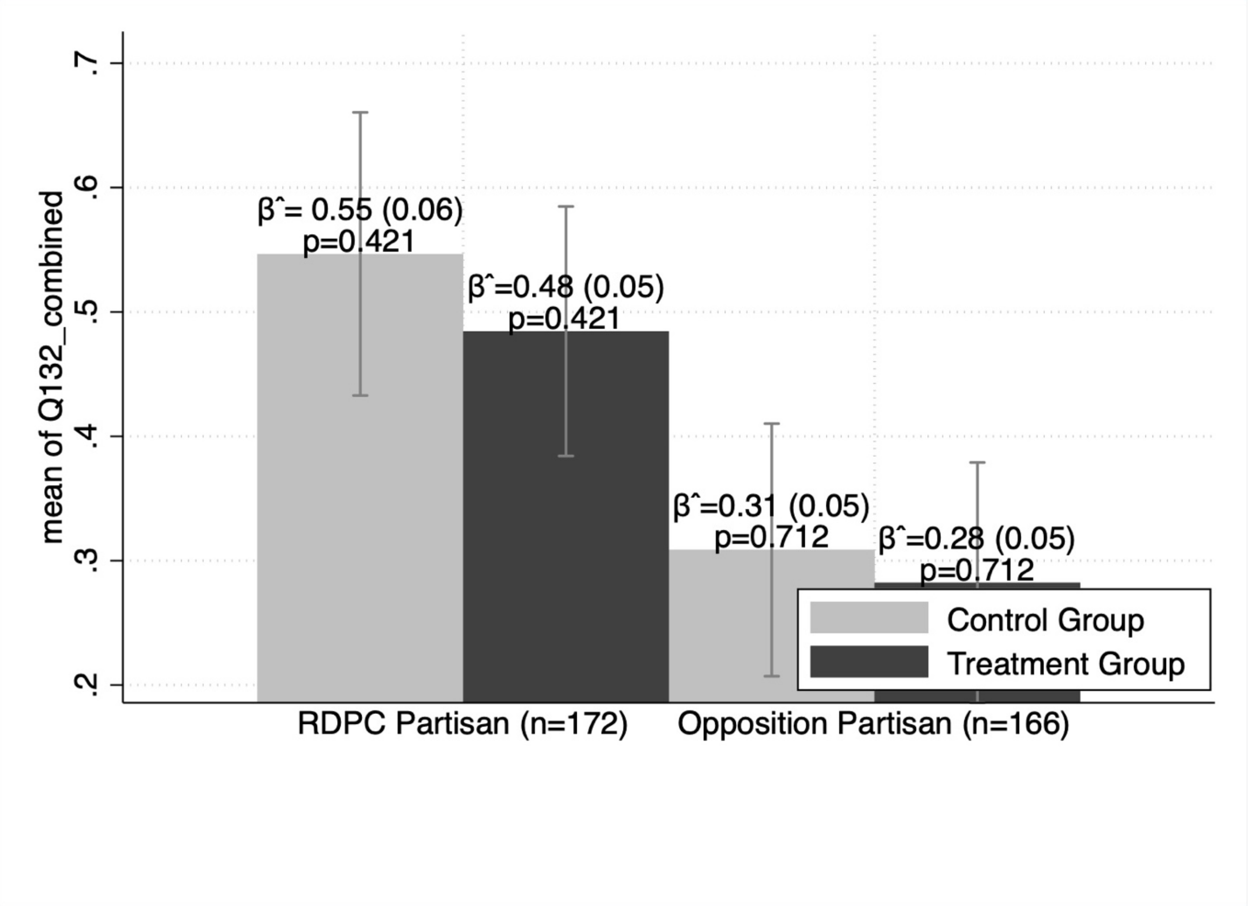

Figure I1 Responses by Treatment Status and Partisanship. *Note*: Mean scores notated with 95% confidence intervals. Standard errors noted in parentheses.

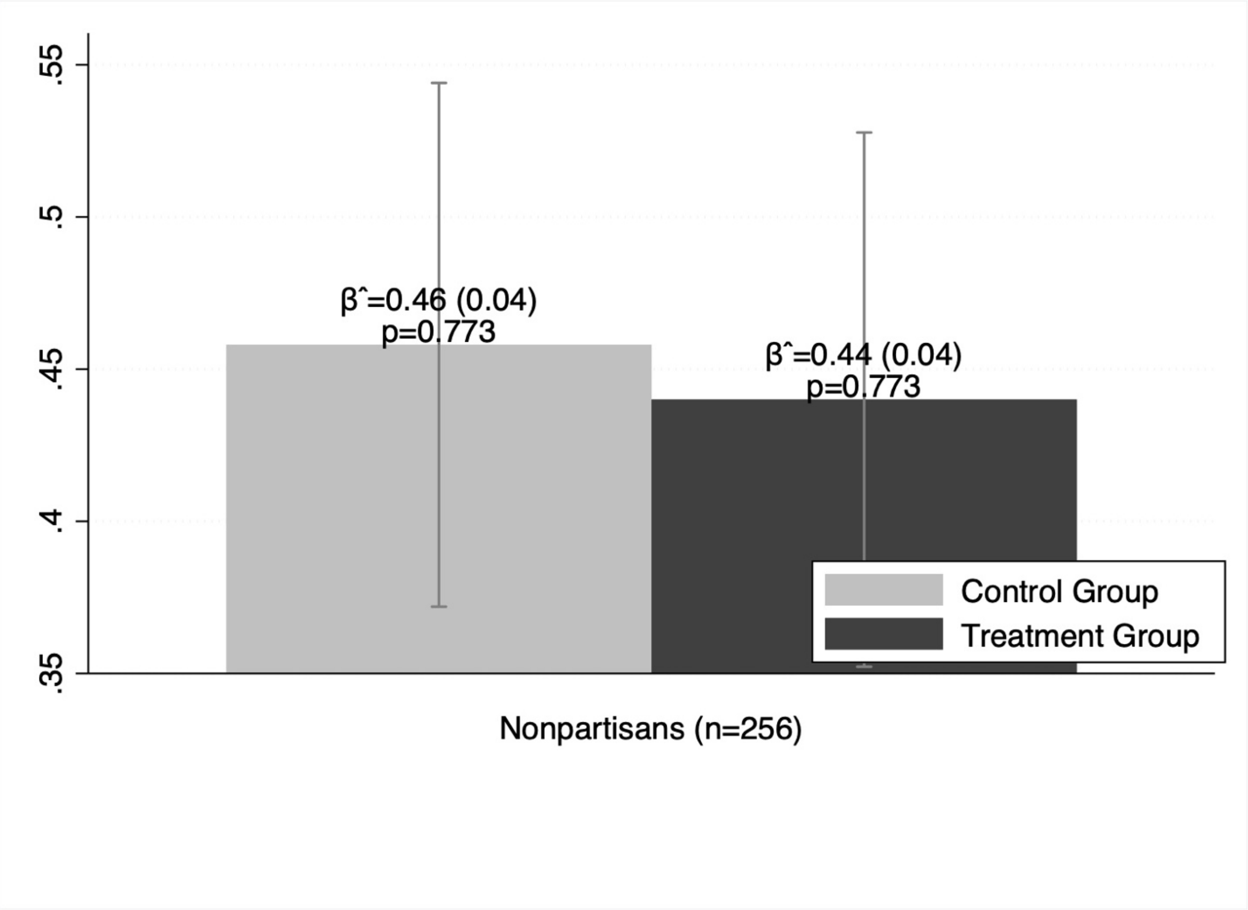

Figure I2 Responses by Treatment Status amongst Nonpartisans. *Note*: Mean scores notated with 95% confidence intervals. Standard errors noted in parentheses.
